# Measles resurgence in Brazil: analysis of the 2019 epidemic in the state of São Paulo

**DOI:** 10.11606/s1518-8787.2022056003805

**Published:** 2022-06-07

**Authors:** Cristina Makarenko, Alexandre San Pedro, Natalia Santana Paiva, Jefferson Pereira Caldas dos Santos, Roberto de Andrade Medronho, Gerusa Gibson

**Affiliations:** I Universidade Federal do Rio de Janeiro Instituto de Estudos em Saúde Coletiva Rio de Janeiro RJ Brasil Universidade Federal do Rio de Janeiro. Instituto de Estudos em Saúde Coletiva. Rio de Janeiro, RJ, Brasil; II Fundação Oswaldo Cruz Escola Nacional de Saúde Pública Sergio Arouca Centro de Estudos, Políticas e Informação sobre Determinantes Sociais da Saúde Rio de Janeiro RJ Brasil Fundação Oswaldo Cruz. Escola Nacional de Saúde Pública Sergio Arouca. Centro de Estudos, Políticas e Informação sobre Determinantes Sociais da Saúde. Rio de Janeiro, RJ, Brasil; III Fundação Oswaldo Cruz Centro de Inovação em Biodiversidade e Saúde Rio de Janeiro RJ Brasil Fundação Oswaldo Cruz. Centro de Inovação em Biodiversidade e Saúde. Rio de Janeiro, RJ, Brasil

**Keywords:** Measles, epidemiology, Disease Transmission, Infectious, Epidemics, Communicable Disease Control, Vaccination Coverage

## Abstract

**OBJECTIVE:**

To analyze the epidemiological profile of cases and the pattern of spatial diffusion of the largest measles epidemic in Brazil that occurred in the post-elimination period in the state of São Paulo.

**METHOD:**

A cross-sectional study based on confirmed measles cases in 2019. Bivariate analysis was performed for socioeconomic, clinical, and epidemiological variables, according to prior vaccination and hospitalization, combined with an analysis of spatial diffusion of cases using the Inverse Distance Weighting (IDW) method.

**RESULTS:**

Of the 15,598 confirmed cases, 2,039 were hospitalized and 17 progressed to death. The epidemic peak occurred in epidemiological week 33, after confirmation of the first case, in the epidemiological week 6. Most cases were male (52.1%), aged between 18 and 29 years (38.7%), identified as whites (70%). Young adults (39.7%) and children under five years (32.8%) were the most affected age groups. A higher proportion of previous vaccination was observed in whites as compared to Blacks, browns, yellows and indigenous people (p < 0.001), as well as in the most educated group compared to the other categories (p < 0.001). The risk of hospitalization was higher in children than in the older age group (RI = 2.19; 95%CI: 1.66–2.88), as well as in the unvaccinated than in the vaccinated (RI = 1.59; 95%CI: 1.45–1.75). The pattern of diffusion by contiguity combined with diffusion by relocation followed the urban hierarchy of the main cities’ regions of influence.

**CONCLUSION:**

In addition to routine vaccination in children, the findings indicate the need for immunization campaigns for young adults. In addition, studies that seek to investigate the occurrence of clusters of vulnerable populations, prone to lower vaccination coverage, are essential to broaden the understanding of the dynamics of transmission and, thus, reorienting control strategies that ensure disease elimination.

## INTRODUCTION

Measles is a highly contagious and life-threatening disease that remains a public health problem, especially in children under five years of age, as well as malnourished and immunosuppressed young adults residing in countries where transmission has not been interrupted^1^.

Since the introduction of mass vaccination in Brazil and worldwide in the 1970s and 1980s, measles cases have decreased substantially. Among the six regions of the World Health Organization, the Americas was the first to have autochthonous transmission recognized as interrupted in 2002 and declared eliminated in 2016, the year in which Brazil was certified as a measles-free country, that is, with the occurrence of less than one case per million in a period of one year^6^.

Despite the advances made in controlling the disease worldwide, the maintenance of endemic transmission of the virus in some countries raises concerns about its re-establishment in regions where the disease had already been considered eliminated. The risk of importing cases and triggering new outbreaks is real, especially in situations where vaccination coverage is less than optimal, posing a challenge for surveillance systems^3,9^.

Recently, many countries in the Americas have reported measles cases, including Brazil, Venezuela, Canada, the United States, Mexico, Peru, and Argentina. In 2018, an outbreak that started in the Northern Region of Brazil, attributed to the D8 genotype imported from Venezuela, culminated in the occurrence of cases that spread to several states, in addition to indigenous communities in Roraima and Amazonas^10,11^. As a result, Brazil lost its status as a measles-free country^12^.

The measles vaccine in Brazil is part of the routine schedule of the National Immunization Program, being offered free of charge in the Unified Health System to the entire population. It is recommended to administer two doses with a minimum interval of 30 days. The first dose of the MMR (measles, mumps and rubella) vaccine should be given at 12 months of age, followed by a booster dose of the tetravalent vaccine (measles, mumps, rubella and varicella) at 15 months. The guarantee of 97% protection is afforded only after the complete vaccination schedule^11,13^.

A sensitive surveillance that effectively responds to the importation of the virus, associated with homogeneous and routine vaccination coverage above 95%, are some of the challenges that countries which have managed to eliminate the disease from their territories have faced^3,9^.

A broad understanding of the profile of those who are acquiring the disease, as well as of its spatial diffusion dynamics is a fundamental requirement to guide actions that guarantee the maintenance of measles elimination^14^. Considering that vaccination is the cornerstone of control efforts, detailed information on cases is essential to assess current vaccination strategies and reorient more targeted interventions, whether through routine immunization services or campaigns targeting specific subpopulations^14^.

This study was designed to analyze the epidemiological profile of measles cases and the pattern of spatial diffusion of measles case in São Paulo state in 2019.

## METHODS

This is a cross-sectional study based on measles cases confirmed by laboratory, clinical and epidemiological criteria, reported in the epidemic year of 2019 in São Paulo state, obtained from the *Sistema de Informação de Agravos de Notificação* (Sinan – Notifiable Diseases Information System).

The choice for São Paulo is due to the fact that the state was the scene of the largest epidemic in Brazil in the post-elimination period, accounting for about 74% of the cases reported in the country in 2019.

São Paulo is the most populous federative unit in the country and the second with the highest Human Development Index. In 2020, its estimated population was 46 million inhabitants spread over an area of 248,209 km^2^. It is located in the southeastern region of the country, and comprises 645 municipalities, distributed in 11 intermediate geographic regions and six metropolitan regions^15^.

For the bivariate descriptive analysis, Pearson’s chi-square test was used for differences in the proportion of previous vaccination of the cases, according to epidemiological, clinical and socioeconomic variables, considering p-value < 0.05 as statistically significant. The hospitalization incidence ratios were also obtained, considering the variables gender, age group, schooling , race/skin color and vaccination status, with the respective intervals with 95% confidence levels.

Epidemiological weeks were considered as units of analysis of the temporal evolution of case notifications. The analyses were performed in the R software, using the EpiR package^16^. Additionally, sequential choropleth maps of the spatial distribution of measles incidence rates were created at municipality level . The rates were smoothed by the Local Empirical Bayesian method, using a neighborhood matrix by contiguity. We opted for a division into equal periods starting from the week of notification of the first case of the year (epidemiological week six). Thus, the periods were defined by the following epidemiological weeks of 2019: from 6 to 18, 19 to 30, 31 to 42, and 43 to 52. Data on population estimates by municipality were obtained from the Brazilian Institute of Geography and Statistics (IBGE).

In the spatial diffusion analysis, we used the cartographic mesh of geographic location of the cities’ headquarters because it is considered that, in some situations, the allocation of centroids by the geometric method may lead to less precision of territory representation, with allocation of points in uninhabited areas. In this way, the use of the cartographic mesh of the municipal headquarters ensures the geographic location of the point within the urban area of the municipality.

The inverse distance weighting (IDW) interpolation method was used to analyze the spatial diffusion of the first confirmed measles cases in each municipality. In this method, sample points are weighted according to the influence of one point relative to another, based on the inverse of the distance. The interpolation process was carried out from the punctual grid of the municipal headquarters, plus information on the dates of onset of symptoms of the first confirmed case and the number of days with confirmed cases notification during the period. The municipalities that did not present confirmed cases during the period received the value of the last date of the series, plus one day. This procedure aimed to ensure that these municipalities were portrayed in the interpolation product as the areas where the spatial spread of measles arrived last or did not arrive until the end of the study period.

We also considered the number of days that each municipality presented new cases as a parameter for interpolation , aiming to emphasize the role of cities that remained as a spreader over time. With this, we sought to mitigate the effect of municipalities that had the notification of the first case at the beginning of the series and the rest of the cases spaced out in time, in contrast to those that notified their first cases on similar dates, but that presented temporal persistence in case notification over the period. The IDW method and the geoprocessing and spatial diffusion mapping techniques were carried out in the QGIS 3.18.1 software.

The study was approved by the research ethics committee of the Collective Health Studies Institute of the Federal University of Rio de Janeiro (IESC/UFRJ) (CAAE protocol nr 4,410,876), with the waiver of free and informed consent, as it uses a secondary database and aggregated analyses, without risk of identifying subjects.

## RESULTS

A total of 15,598 measles cases were confirmed in the state of São Paulo in 2019, of which 2,039 were hospitalized and 17 died. Approximately 40% of confirmed cases (6,302) reported prior measles vaccination. The first case, a 6-month-old child residing in the city of São Paulo, was reported on February 7, 2019, in epidemiological week (EW) six. The peak of transmission occurred six months later, in August (EW 33), when 5,539 cases were reported in 12.7% of the municipalities of São Paulo state, of which 759 (13.5%) cases were hospitalized and five died (Table 1).

**Table 1:** Monthly frequency of notifications of confirmed, vaccinated, hospitalized cases and deaths in the state of São Paulo, 2019.

Notification month	Total cases	Imported cases^a^ (%)	Vaccinated(%)	Hospitalized (%)	Deaths	Affected municipalities(%)
Jan	0	0	0	0	0	0
Feb	5	0	4 (80.0)	0	0	3 (0.5)
Mar	11	0	4 (36.4)	2 (18.2)	0	2 (0.3)
Apr	24	1 (4.2)	12 (50.0)	2 (8.3)	0	7 (1.1)
May	57	2 (5.3)	24 (42.1)	10 (17.5)	0	11 (1.7)
Jun	460	0	198 (43.0)	59 (12.8)	0	34 (5.3)
Jul	2,957	4 (0.1)	1,199 (40.5)	373 (12.6)	2	101 (15.7)
Aug	5,539	11 (0.2)	2,237 (40.4)	759 (13.7)	5	175 (27.1)
Sept	3,765	8 (0.2)	1,502 (39.9)	467 (12.4)	8	178 (27.6)
Oct	1,429	3 (0.2)	571 (40.0)	189 (13.2)	1	139 (21.6)
Nov	843	3 (0.4)	338 (40.1)	111 (13.2)	0	96 (14.9)
Dec	508	1 (0.2)	213 (41.9)	67 (13.2)	1	74 (11.5)

Source: Sinan, Datasus, 2019.

^a^ Cases imported from other states or countries.

**Figure 1:**
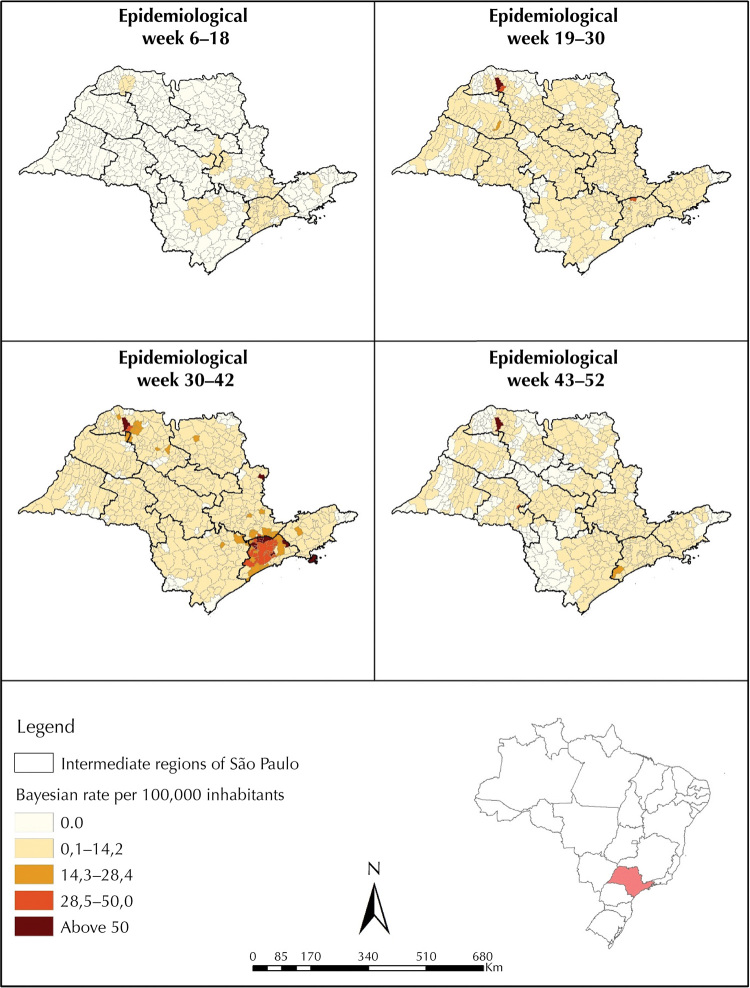
Evolution of measles incidence rates per 100,000 inhabitants by municipalities in the state of São Paulo during the 2019 epidemic year.

Source: Sinan, Datasus.

Regarding the spatial evolution of incidence rates over epidemiological weeks, the highest were observed in municipalities located in the Intermediate Region of São Paulo, especially in those that make up the metropolitan region. A high incidence was also observed in Fernandópolis, a municipality located in the northwest of the state, which might be due to the unstable rates arising from small populations, although they have been smoothed (Figure 1).

Over the period, 33 cases imported from outside the state of São Paulo were identified, of which 20 were from other federative units and 13 from other countries, such as Ivory Coast, Ghana and Bangladesh. The first four cases of the epidemic were classified as autochthonous in the city of São Paulo and were reported in February, followed by notification in surrounding cities. Cases classified as imported began to be reported two months (April) after the first case appeared, the first of which came from Minas Gerais State. The other cases classified as imported were registered in the following months, especially in the months with the highest number of notifications (July, August and September) (Table 1).

**Figure 2:**
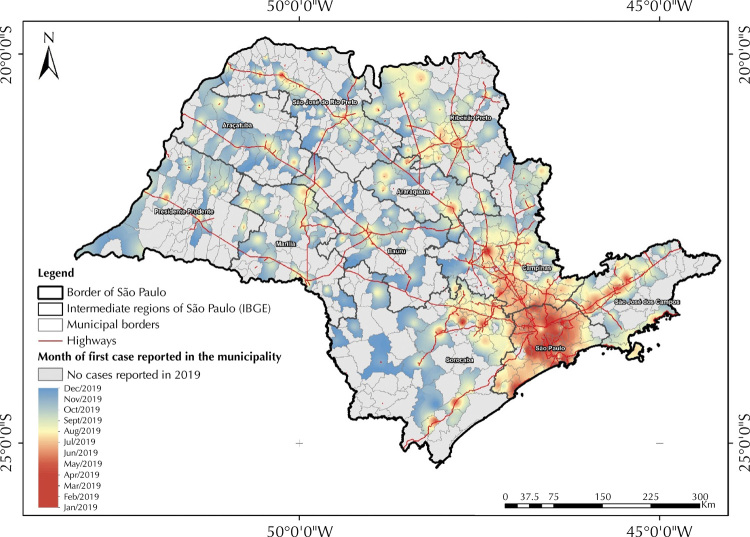
Diffusion of measles cases in the state of São Paulo, according to the date of onset of the first symptoms.

Source: Sinan, Datasus.

Considering the date of onset of symptoms, the first cases of 2019 were reported in the municipalities of São Bernardo do Campo and São Paulo on the dates of 01/01/2019 and 01/08/2019, respectively. Both municipalities are part of the intermediate region called São Paulo, which corresponds to the metropolitan regions of São Paulo and Baixada Santista. This region was the pioneer and main hub for the spread of measles throughout the state. In the subsequent four months, a radial (center-peripheral) spread of the disease was observed within the region itself. Subsequently, this diffusion takes place mainly in three main axes: the most expressive axis is the one that goes towards the intermediate region of Campinas, followed by the axis that goes towards the intermediate region of São José dos Campos (Vale do Paraíba) and the last one in the northeast direction of the intermediate region of Sorocaba (Figure 2).

Finally, two other diffusion poles of lesser magnitude were also observed, the first formed by the municipalities of Ribeirão Preto, Dumont, Pitangueiras, Sertãozinho, Barrinha, Pontal and Jardinópolis, located in the intermediate region of Ribeirão Preto, and the second located in the intermediate region of São José do Rio Preto. The last city to report measles cases in 2019 considering the onset of the first symptoms (12/15/2019) was the municipality of Eldorado (Figure 2).

Of the 15,598 confirmed cases in 2019, 52.1% were male (8,123) and 47.9% were female (7,471). The most affected age group was 18–29 years old (39.7%; 6,190), followed by children under five years old (32.8%; 5,124). Among those over 18 years old, 56.1% (1,987) had between 10 and 12 years of formal schooling, 24.1% (852) had between four and nine years of schooling, 14.2% (504) had 13 years or more of schooling. The less educated group (less than four years of schooling) corresponded to 5.6% (198) of cases aged over 18 years (Table 2).

**Table 2:** Epidemiological and socioeconomic characteristics of measles cases, stratified according to previous vaccination. State of São Paulo, Brazil, 2019.

Variables	Frequency^a^		Prior vaccination		p
	n	%	Yes^b^	No^b^	
Gender					< 0.001
Male	8,123	52.1	3,074 (37.8)	3,380 (41.6)	
Female	7,471	47.9	3,228 (43.2)	2,728 (36.5)	
Age group					< 0.001
< 1 year	2,841	18.2	1,112 (39.1)	1,050 (36.9)	
1–4 years	2,283	14.6	964 (42.2)	879 (38.5)	
5–11 years	499	3.2	222 (44.4)	192 (38.4)	
12–17 years	677	4.3	300 (44.3)	226 (33.3)	
30–49 years	2,678	17.2	994 (37.1)	1,048 (39.1)	
18–29 years	6,190	39.7	2,556 (41.2)	2,579 (41.6)	
50 years or older	430	2.8	154 (35.8)	205 (47.6)	
Schooling^c^					< 0.001
< 4 years	198	5.6	87 (43.9)	85 (42.9)	
4–9 years	852	24.1	281 (32.9)	438 (51.4)	
10–12 years	1,987	56.1	695 (34.9)	947 (47.6)	
13 years or more	504	14.2	224 (44.4)	188 (37.3)	
Race/skin color					< 0.001
White	9,246	70.7	4,046 (43.7)	3,566 (38.5)	
Black	427	3.3	156 (36.5)	198 (46.7)	
Brown	3,241	24.8	1,195 (36.8)	1,505 (46.4)	
Yellow	130	1.0	53 (40.7)	53 (40.7)	
Indigenous	37	0.3	14 (37.8)	18 (48.6)	
Source of transmission^d^					0.02
Imported	33	0.3	20 (60.6)	10 (30.3)	
Autochthonous – ESP	12,832	99.7	5,308 (41.3)	5,076 (39.5)	
Evolution					0.04
Cure	13,019	99.9	5,387 (41.3)	5,225 (40.1)	
Death	17	0.1	3 (17.6)	10 (58.8)	

Source: Sinan, 2019.

^a^ Cases with unknown information were excluded.

^b^ Absolute and relative frequencies.

^c^ Only cases aged 18 and over.

^d^ Autochthonous in the state of São Paulo, SP were considered.

Whites made up the largest share of measles cases, equivalent to 70.7% (9,246), followed by browns 24.8% (3,241), Blacks 3.3% (427), yellows (Asians) 1% (130), and indigenous people 0.3% (37) (Table 2).

Most cases (12,832; 99.9%) were autochthonous from São Paulo State. Among imported cases, most (20/33) came from other states in the country, with the remaining (13/33) being international travelers (Table 2).

A significant difference (p < 0.001) was observed in the proportions of previous vaccination between female (43.2%) and male (37.9%) cases. Regarding age, the frequencies of previous vaccination were higher in the younger than in the older groups (p < 0.001). Regarding education, the largest proportions of previous vaccination were observed in the two extreme categories, that is, in the group with less than four years of schooling and in the group with more than 13 years of schooling (p < 0.001) (Table 2).

In relation to race/skin color, a higher share of vaccination was observed among whites (43.7%) as compared to Blacks (36.5%), browns (36.8%), yellows (40.7%) and indigenous people (37.8%) (p < 0.001). Similarly, a higher frequency of previous vaccination was observed among imported (60.6%) as compared to autochthonous cases (41.3%) (p = 0.02), as well as among those that progressed to cure (41.3%) compared to those who progressed to death (17.6%) (p = 0.04) (Table 2).

In addition to fever and skin rash, cough and runny nose were the most frequent signs and were present in 79.1% (11,871) and 65.4% (9,779) of the cases, respectively. On the other hand, conjunctivitis was less frequent and was present in 34.9% (5,144) of confirmed measles cases in 2019. When stratified by vaccination status, cough and conjunctivitis were significantly more frequent among unvaccinated cases compared to cases that reported previous vaccination (p < 0.001).

Regarding the incidence of hospitalization, there was a higher risk (IR = 2.19; 95%CI: 1.66–2.88) in children under one year of age than in the older age group (50 years and over) (Table 3). Additionally, a higher risk of hospitalization was observed in unvaccinated cases than in those who reported previous vaccination (IR = 1.59; 95%CI: 1.45–1.75) (Table 3).

**Table 3:** Incidence of hospitalization according to epidemiological and socioeconomic characteristics of measles cases. State of São Paulo, Brazil, 2019.

Variables	Total cases^a^	Hospitalization		IR	95%CI
		n	incidence^a^		
Gender					
Male	8,123	1,086	13.4	1	
Female	7,471	952	12.7	0.95	(0.87–1.03)
Age group					
50 or older	430	47	10.9	1	
30–49	2,678	279	10.4	1.03	(0.77–1.38)
18–29	6,190	601	9.7	0.90	(0.68–1.19)
12–17	677	68	10.0	0.92	(0.65–1.30)
5–11	499	50	10.0	0.93	(0.64–1.36)
1–4	2,283	304	13.3	1.22	(0.92–1.63)
< 1	2,841	690	24.3	2.19	(1.66–2.88)
Schooling^b^					
< 4 years	198	10	5.1	1	
4–9 years	852	44	5.2	1.07	(0.55–2.08)
10–12 years	1,987	118	5.9	1.20	(0.64–2.24)
13 years or more	504	45	8.9	1.14	(0.58–2.25)
Registered race/color					
White	9,246	1,168	12.6	1	
Black	427	44	10.3	0.82	(0.62–1.09)
Brown	3,241	389	12.0	0.96	(0.87–1.07)
Yellow	130	17	13.1	1.01	(0.64–1.56)
Indigenous	37	8	21.6	1.84	(1.01–3.34)
Prior vaccination					
Yes	6,302	642	10.2	1	
No	6,179	1,004	16.2	1.59	(1.45–1.75)

Source: Sinan, 2019.

IR: incidence rate; 95%CI: 95% confidence interval.

^a^ Cases with unknown information were excluded.

^b^ Only cases aged 18 or over.

## DISCUSSION

The findings of the present study provide important reflections on the largest measles epidemic in Brazil in the post-elimination period. Regarding the origin of the epidemic, secondary data did not allow a definitive conclusion. Although there is no detail on the origin of the first case reported in the state of São Paulo in 2019, it is assumed that it is not the index case, as it involves a six-month-old infant, classified as autochthonous. The first cases of an outbreak in areas that have eliminated endemic transmission of measles virus can be confused with other non-specific exanthematous viral infections. In addition, clinical manifestations in vaccinated individuals (although with incomplete schedules) tend to be milder, which can lead to underreporting^9,17,18^.

The findings of the present study corroborate this fact by indicating a higher risk of hospitalization among unvaccinated than among vaccinated cases, as well as the differences observed in the clinical presentation of the disease, with a lower frequency of cough and conjunctivitis in vaccinated cases as compared to those with no vaccination history.

The considerable number of cases in individuals with a history of previous vaccination is a worrying fact that may suggest the vaccine’s loss of effectiveness or even limitations in the recording of this information in the notification systems. Some studies have reported the development of the disease in people who were vaccinated with both doses in childhood. Some possible reasons include the reduction of antibody levels over time and loss of effectiveness due to cold chain issues^19^. There is also evidence that differences between the genotypes of the virus used in the vaccine and the genotypes of the circulating strains may also contribute to the development of the disease in vaccinated individuals^23,24^.

Regarding the quality of the record, although there is technical guidance on checking the vaccination book of cases, it is more likely that this information in the routine of care services is self-reported. Additionally, it is important to note that the notification form does not specify whether the case had a complete vaccination schedule with two doses. Thus, it is possible that part of the cases in individuals who reported prior vaccination in the São Paulo epidemic had incomplete vaccination schedules, with a consequent reduction in vaccine effectiveness. The greater involvement of young adults in the São Paulo epidemic reinforces this hypothesis, since this age group has been identified in other studies as the most likely to have an incomplete vaccination schedule^4,25^.

Children under five years of age were the second age group most affected by the disease and, together with young adults, made up the predominant age profile of the São Paulo epidemic. This finding corroborates the trend of increased measles morbidity in young adults, previously restricted to children^4^.

If, on the one hand, there may be some loss in vaccination effectiveness or even incomplete vaccination schedules, on the other hand, the analysis of the data also pointed to a significant number of cases without prior vaccination, which, in any case, indicates low performance of the routine immunization programs or inadequacy in recording this information. The formation of clusters of susceptible populations resulting from inadequate vaccine coverage can act as initiators of outbreaks in areas where the disease is being eliminated, as demonstrated in previous studies^9,18^.

In children, there is a tendency for vaccine coverage to decrease with increasing age, especially in relation to vaccines administered in multiple doses, such as the MMR. In the first year of life, the child receives a large number of vaccines from the routine schedule, followed by an interval of months or years, which generates a sense of tranquility that can contribute to forgetfulness by parents and caregivers^31,32^. It is important to remember that in Brazil, the second dose of MMR, which was previously recommended at 4–6 years, was brought forward to 15 months of age in 2013^33^. This fact may explain, at least in part, the higher frequency of cases in young adults and in older birth cohorts. It is worth remembering that the measles vaccine is recommended up to 49 years of age, as it is assumed that above that age the person has already had natural contact with the virus, thus having lasting immunity.

The findings also revealed a lower proportion of vaccination in less educated groups compared to those with higher education (13 or more years of schooling), as well as in Black and brown individuals compared to whites, which suggest unequal access to vaccination^34^. The fact that 70% of the cases were identified as whites may be related to the greater purchasing power and possibilities of traveling to places with virus circulation of this group, and, as a consequence, a greater likelihood of infection of close people on return.

As for the spatial diffusion analysis, the findings revealed two main patterns. The first, characterized by a process of diffusion by contiguity, in which from the pioneer pole (metropolitan regions of São Paulo and Baixada Santista) three main axes were established that connect nearby municipalities located in the intermediate regions of Campinas, São José dos Campos and Sorocaba. The second pattern is characterized by the relationship between the pioneer hub and more distant municipalities, belonging to the intermediate regions of Ribeirão Preto and São José do Rio Preto, through a process of hierarchical diffusion by relocation.

Both patterns of measles diffusion reflect the structural characteristics of the territory, following trajectories similar to those of the main highways in the state and the urban hierarchy of cities’ regions of influence in relation to the centrality in the distribution of goods, equipment, services and population mobility. In this regard, the role of the greater São Paulo (a national metropolis), the regional centers Campinas, São José dos Campos, Sorocaba, Ribeirão Preto, Santos, Limeira, and the sub-regional centers Itapetinga, Fernandópolis and Registro is highlighted. In addition to being a pioneer hub and main spreader of cases to other regions, it is worth noting that the Greater São Paulo region maintained high incidences during most of the epidemic in 2019.

These findings reinforce the importance of urban hierarchy in the process of spreading diseases with transmission similar to measles’, as demonstrated in recent studies carried out for covid-19 in the states of São Paulo^35^and Rio de Janeiro^36^. The incorporation of other information on the territory not used in this study, such as the flows of people on air and road routes, can contribute to a better characterization of the processes involved in the spread of measles and support surveillance actions at the intercity level.

The limitations of this study are mainly related to the quality of the information coming from the information system. In the case of previous vaccination records, the information is likely to be self-reported in most cases, despite the guidance to check the vaccination record book. As for the race/skin color variable, although it is a field in the notification form intended for self-identification, it is relatively common in the routine of care services for it to be filled in by the professional who attended to the case. In this sense, depending on the perceptions of those who collect this information, the measurement of access inequities based on this variable can be biased.

Ultimately, the results reinforce the importance of prioritizing immunization strategies for the most affected subgroups, formed by young adults, in addition to routine vaccination in children. Additionally, studies that seek to investigate the occurrence of clusters of vulnerable populations, prone to lower vaccination coverage, are essential to broaden the understanding of the dynamics of disease transmission and thus reorient more effective control strategies to ensure the maintenance of the elimination of the disease.
