# Supplementary material for: Measles resurgence in Brazil: analysis of the 2019 epidemic in the state of São Paulo
Source: Rev Saude Publica. 2022 Jun 7;56:50. doi: 10.11606/s1518-8787.2022056003805 (PMC9239333; doi:10.11606/s1518-8787.2022056003805)
Supplement: Translation

# Translation: Ressurgimento do sarampo no Brasil: análise da epidemia de 2019 no estado de São Paulo

**Authors:** Cristina Makarenko, Alexandre San Pedro, Natalia Santana Paiva, Jefferson Pereira Caldas dos Santos, Roberto de Andrade Medronho, Gerusa Gibson

**Affiliations:** I Brasil Universidade Federal do Rio de Janeiro. Instituto de Estudos em Saúde Coletiva. Rio de Janeiro, RJ, Brasil; II Brasil Fundação Oswaldo Cruz. Escola Nacional de Saúde Pública Sergio Arouca. Centro de Estudos, Políticas e Informação sobre Determinantes Sociais da Saúde. Rio de Janeiro, RJ, Brasil; III Brasil Fundação Oswaldo Cruz. Centro de Inovação em Biodiversidade e Saúde. Rio de Janeiro, RJ, Brasil

**Keywords:** Sarampo, epidemiologia, Transmissão de Doença Infecciosa, Epidemias, Controle de Doenças Transmissíveis, Cobertura Vacinal

## Abstract

**OBJETIVO:**

Analisar o perfil epidemiológico dos casos e o padrão de difusão espacial da maior epidemia de sarampo do Brasil ocorrida no período pós-eliminação, no estado de São Paulo.

**MÉTODO:**

Estudo transversal, baseado em casos confirmados de sarampo em 2019. Foi conduzida análise bivariada das variáveis socioeconômicas, clínicas e epidemiológicas, segundo vacinação prévia e ocorrência de hospitalização, combinada a uma análise de difusão espacial dos casos por meio da metodologia de interpolação pela ponderação do inverso da distância.

**RESULTADOS:**

Dos 15.598 casos confirmados, 2.039 foram hospitalizados e 17 evoluíram para o óbito. O pico epidêmico ocorreu na semana epidemiológica 33, após a confirmação do primeiro caso, na semana epidemiológica 6. A maioria dos casos era homem (52,1%), com idade entre 18 e 29 anos (38,7%), identificados como brancos (70%). Adultos jovens (39,7%) e menores de cinco anos (32,8%) foram as faixas etárias mais acometidas. Observou-se maior proporção de vacinação prévia em brancos, quando comparados a pretos, pardos, amarelos e indígenas (p < 0,001), assim como no grupo mais escolarizado, quando comparado às demais categorias (p < 0,001). O risco de hospitalização foi maior em crianças, quando comparado à faixa etária mais idosa (RI = 2,19; IC95% 1,66–2,88), assim como entre não vacinados, quando comparado a vacinados (RI = 1,59; IC95% 1,45–1,75). O padrão de difusão por contiguidade combinado à difusão por realocação seguiu a hierarquia urbana das regiões de influência das principais cidades.

**CONCLUSÃO:**

Além da vacinação de rotina em crianças, os achados indicam a necessidade de campanhas de imunização de adultos jovens. Adicionalmente, estudos que busquem investigar a ocorrência de *clusters* de populações vulneráveis, propensas a menor cobertura de vacinação, são essenciais para ampliar a compreensão sobre a dinâmica de transmissão da doença e, assim, reorientar estratégias de controle que garantam a eliminação da doença.

## INTRODUÇÃO

O sarampo é uma doença altamente contagiosa e potencialmente fatal que permanece como um problema de saúde pública, especialmente em crianças menores de cinco anos, assim como adultos jovens desnutridos e imunodeprimidos residentes de países em que a transmissão não foi interrompida^1^.

Desde a introdução da vacinação em massa no Brasil e no mundo nas décadas de 1970 e 1980, os casos de sarampo diminuíram substancialmente. Dentre as seis regiões da Organização Mundial da Saúde, a das Américas foi a primeira a ter transmissão autóctone reconhecida como interrompida em 2002 e declarada eliminada em 2016, ano em que o Brasil recebeu a certificação de país livre do sarampo, ou seja, quando constatada a ocorrência de menos de um caso por milhão no período de um ano^6^.

Apesar dos avanços alcançados no controle da doença em todo o mundo, a manutenção da transmissão endêmica do vírus em alguns países gera preocupação quanto ao seu restabelecimento em regiões onde a doença já fora considerada eliminada. O risco de importação de casos e desencadeamento de novos surtos é real, especialmente em situações em que as coberturas vacinais estão abaixo do ideal, configurando-se como um desafio para os sistemas de vigilância^3,9^.

Recentemente, muitos países das Américas notificaram a ocorrência de casos, incluindo Brasil, Venezuela, Canadá, Estados Unidos, México, Peru e Argentina. Em 2018, um surto iniciado na Região Norte do Brasil, atribuído ao genótipo D8, importado da Venezuela, culminou na ocorrência de casos que se disseminaram por vários estados, além de comunidades indígenas localizadas em Roraima e no Amazonas^10,11^. Como consequência, o Brasil perdeu o título de país livre do sarampo^12^.

A vacina contra o sarampo no Brasil faz parte do calendário de rotina do Programa Nacional de Imunizações, sendo ofertada gratuitamente no Sistema Único de Saúde a toda população. Recomenda-se a aplicação de duas doses com intervalo mínimo de 30 dias. A primeira dose da vacina tríplice viral (sarampo, caxumba e rubéola) deve ser aplicada aos 12 meses de vida, seguida de uma dose de reforço da vacina tetra viral (sarampo, caxumba, rubéola e varicela) aos 15 meses, sendo que a garantia de 97% de proteção ocorre somente após o esquema completo^11,13^.

A existência de uma vigilância sensível que responda de forma eficaz à importação do vírus, associada a coberturas vacinais homogêneas e de rotina acima de 95%, são alguns dos desafios que os países que conseguiram eliminar a doença dos seus territórios enfrentam^3,9^.

A ampla compreensão do perfil de quem está adquirindo a doença, assim como da sua dinâmica de difusão espacial é um requisito fundamental para orientar ações que garantam a manutenção da eliminação do sarampo^14^. Considerando que a vacinação é a base dos esforços de controle, informações detalhadas sobre os casos são essenciais para avaliar as estratégias de vacinação atuais e reorientar intervenções mais direcionadas, seja por meio dos serviços de imunização de rotina ou de campanhas orientadas a subpopulações específicas^14^.

O objetivo do presente estudo foi analisar o perfil epidemiológico dos casos de sarampo e o padrão de difusão espacial da epidemia no estado de São Paulo em 2019.

## MÉTODOS

Trata-se de um estudo transversal baseado nos casos de sarampo confirmados por critério laboratorial, clínico e vínculo epidemiológico, notificados no ano epidêmico de 2019, no estado de São Paulo, obtidos do Sistema de Informação de Agravos de Notificação (Sinan).

A escolha por São Paulo se dá devido ao fato de o estado ter sido cenário da maior epidemia do Brasil no período pós-eliminação, concentrando cerca de 74% dos casos notificados no país em 2019.

São Paulo é a unidade federativa mais populosa do país e a segunda com maior Índice de Desenvolvimento Humano. Em 2020 sua população estimada era de 46 milhões de habitantes distribuídos em uma área de 248.209 km^2^. Localiza-se na região sudeste do país, e é formado por 645 municípios, distribuídos em 11 regiões geográficas intermediárias e seis regiões metropolitanas^15^.

Para a análise descritiva bivariada foi utilizado o teste qui-quadrado de Pearson para as diferenças de proporção de vacinação prévia dos casos, conforme as variáveis epidemiológicas, clínicas e socioeconômicas, considerando estatisticamente significativo os valores de p < 0,05. Foram também obtidas as razões de incidência de hospitalização, considerando as variáveis, sexo, faixa etária, escolaridade, raça/cor da pele e situação vacinal, com os respectivos intervalos com níveis de confiança de 95%.

As semanas epidemiológicas foram consideradas como unidades de análise da evolução temporal das notificações de casos. As análises foram realizadas no *software* R, usando o pacote EpiR^16^. Adicionalmente, foram elaborados mapas coropléticos sequenciais da distribuição espacial das taxas de incidência de sarampo, por municípios do estado de São Paulo. As taxas foram suavizadas pelo método Bayesiano Empírico Local, utilizando matriz de vizinhança por contiguidade. Optou-se por uma divisão em períodos iguais a partir da semana de notificação do primeiro caso do ano (semana epidemiológica seis). Dessa forma, os períodos foram definidos pelas seguintes semanas epidemiológicas de 2019: de seis a 18, de 19 a 30, 31 a 42, da 43 a 52. Os dados das estimativas populacionais por município foram obtidos do Instituto Brasileiro de Geografia e Estatística (IBGE).

Na análise da difusão espacial, foi utilizada a base cartográfica de localização geográfica das sedes dos municípios por se considerar que, em algumas situações, a alocação dos centroides pelo método geométrico pode resultar na perda de precisão da representação do território, com alocação do ponto em áreas não habitadas. Dessa maneira, a utilização da malha cartográfica das sedes municipais garante a localização geográfica do ponto dentro da área urbana do município.

A metodologia de interpolação pela ponderação do inverso da distância (IDW, do inglês *inverse distance weighting*) foi utilizada para análise da difusão espacial dos primeiros casos confirmados de sarampo em cada município. Nesse método, as amostras de pontos são ponderadas durante a interpolação de acordo com a influência de um ponto relativo a outro, baseada no inverso da distância. O processo de interpolação foi realizado a partir da malha pontual das sedes dos municípios do estado de São Paulo, acrescida das informações das datas de início de sintomas do primeiro caso confirmado e do número de dias com notificação de casos confirmados durante o período. Os municípios que não apresentaram casos confirmados durante o período analisado receberam o valor da última data da série, acrescida de um dia. Esse procedimento visou garantir que esses municípios fossem retratados no produto da interpolação como as áreas onde a difusão espacial de sarampo chegou por último ou não chegou até o fim do período do estudo.

Outro procedimento realizado foi a utilização do número de dias que o município apresentou novos casos como parâmetro na interpolação, visando enfatizar o papel das cidades que se mantiveram na função difusora ao longo do tempo. Com isso, buscou-se mitigar o efeito de municípios que apresentaram a notificação do primeiro caso no início da série e o restante dos casos espaçados no tempo, em contraposição àqueles que notificaram seus primeiros casos em datas semelhantes, mas que apresentaram persistência temporal na notificação de casos ao longo do período. A metodologia IDW e as técnicas de geoprocessamento e mapeamento da difusão espacial foram realizadas no programa QGIS 3.18.1.

O estudo foi aprovado pelo comitê de ética em pesquisa do Instituto de Estudos em Saúde Coletiva da Universidade do Rio de Janeiro (IESC/UFRJ), sob parecer número 4.410.876, com dispensa de termo de consentimento livre e esclarecido, por empregar base de dados secundários e análises agregadas, sem risco de identificação dos sujeitos.

## RESULTADOS

Foram confirmados 15.598 casos de sarampo no estado de São Paulo no ano de 2019, dos quais 2.039 foram hospitalizados e 17 evoluíram para óbito. Aproximadamente 40% dos casos confirmados (6.302) relataram vacinação prévia para sarampo. O primeiro caso, uma criança de 6 meses residente da cidade de São Paulo, foi notificado no dia 7 de fevereiro de 2019, na semana epidemiológica (SE) seis. O pico de transmissão ocorreu seis meses depois, no mês de agosto (SE 33), quando 5.539 casos foram notificados em 12,7% dos municípios do estado, dos quais 759 (13,5%) casos foram hospitalizados e cinco evoluíram para óbito (Tabela 1).

**Tabela 1:** Frequência mensal das notificações de casos confirmados, vacinados, hospitalizados e óbitos no estado de São Paulo, 2019.

Mês de notificação	Total de casos	Casos^a^ importados (%)	Vacinados (%)	Hospitalizados (%)	Óbitos	Municípios afetados (%)
Jan	0	0	0	0	0	0
Fev	5	0	4 (80,0)	0	0	3 (0,5)
Mar	11	0	4 (36,4)	2 (18,2)	0	2 (0,3)
Abr	24	1 (4,2)	12 (50,0)	2 (8,3)	0	7 (1,1)
Mai	57	2 (5,3)	24 (42,1)	10 (17,5)	0	11 (1,7)
Jun	460	0	198 (43,0)	59 (12,8)	0	34 (5,3)
Jul	2.957	4 (0,1)	1.199 (40,5)	373 (12,6)	2	101 (15,7)
Ago	5.539	11 (0,2)	2.237 (40,4)	759 (13,7)	5	175 (27,1)
Set	3.765	8 (0,2)	1.502 (39,9)	467 (12,4)	8	178 (27,6)
Out	1.429	3 (0,2)	571 (40,0)	189 (13,2)	1	139 (21,6)
Nov	843	3 (0,4)	338 (40,1)	111 (13,2)	0	96 (14,9)
Dez	508	1 (0,2)	213 (41,9)	67 (13,2)	1	74 (11,5)

Fonte: Sinan, Datasus, 2019.

^a^ Casos importados de outros estados ou países.

Em relação à evolução espacial das taxas de incidência ao longo das semanas epidemiológicas, as maiores foram observadas em municípios situados na Região Intermediária de São Paulo, em especial naqueles que compõem a região metropolitana. Foi observada também elevada incidência no município de Fernandópolis, no noroeste do estado, fato que se deve à instabilidade das taxas estimadas para populações pequenas, ainda que tenham sido suavizadas (Figura 1).

**Figura 1:** Evolução das taxas de incidências de sarampo por 100.000 habitantes por municípios do estado de São Paulo ao longo do ano epidêmico de 2019.

Fonte: Sinan, Datasus.

Ao longo do período foram identificados 33 casos importados de fora do estado de São Paulo, dos quais 20 eram procedentes de outras unidades federativas e 13 de outros países, como Costa do Marfim, Gana e Bangladesh. Os quatro primeiros casos da epidemia foram classificados como autóctones na cidade de São Paulo e foram notificados em fevereiro, seguido da notificação em cidades do entorno. Os casos classificados como importados começaram a ser notificados após dois meses (abril) do surgimento do primeiro caso, sendo o primeiro deles procedente de Minas Gerais. Os demais casos classificados como importados foram registrados nos meses seguintes, em especial nos meses de maior pico de notificações (julho, agosto e setembro) (Tabela 1).

Considerando a data de início dos sintomas, os primeiros casos de 2019 foram notificados nos municípios de São Bernardo do Campo e em São Paulo nas datas de 01/01/2019 e 08/01/2019 respectivamente. Ambos os municípios fazem parte da região intermediária denominada de São Paulo, que corresponde às regiões metropolitanas de São Paulo e Baixada Santista. Essa região foi o polo pioneiro e principal de difusão de sarampo pelo estado. Nos quatro meses subsequentes, observou-se uma difusão radial (centro-periférica) da doença dentro da própria região. Posteriormente essa difusão se faz majoritariamente em três eixos principais: o eixo mais expressivo é o que vai na direção da região intermediária de Campinas, seguido do eixo que segue na direção da região intermediária de São José dos Campos (Vale do Paraíba) e do último na direção nordeste da região intermediária de Sorocaba (Figura 2).

**Figura 2:** Difusão dos casos de sarampo no estado de São Paulo, segundo data de aparecimento dos primeiros sintomas.

Fonte: Sinan, Datasus.

Por fim, também foram observados dois outros polos de difusão de menor magnitude, o primeiro formado pelos municípios de Ribeirão Preto, Dumont, Pitangueiras, Sertãozinho, Barrinha, Pontal e Jardinópolis, localizados na região intermediária de Ribeirão Preto, e o segundo localizado na região intermediária de São José do Rio Preto. A última cidade a notificar casos de sarampo em 2019 considerando o início dos primeiros sintomas (15/12/2019) foi o município de Eldorado (Figura 2).

Dos 15.598 casos confirmados em 2019, 52,1% eram do sexo masculino (8.123) e 47,9% do sexo feminino (7.471). A faixa etária mais acometida foi a de 18 a 29 anos (39,7%; 6.190), seguida da de crianças menores de cinco anos (32,8%; 5.124). Dentre os maiores de 18 anos de idade, 56,1% (1.987) tinham entre 10 e 12 anos de estudo formal, 24,1% (852) tinham entre quatro e nove anos de estudo, 14,2% (504) tinham 13 anos ou mais de estudo. O grupo com menor escolaridade (menos de quatro anos de estudo) correspondeu a 5,6% (198) dos casos com idade acima de 18 anos (Tabela 2).

**Tabela 2:** Características epidemiológicas e socioeconômicas dos casos de sarampo, estratificadas segundo vacinação prévia. Estado de São Paulo, Brasil, 2019.

Variáveis	Frequência^a^		Vacinação prévia		p
	n	%	Sim^b^	Não^b^	
Sexo					< 0,001
Masculino	8.123	52,1	3.074 (37,8)	3.380 (41,6)	
Feminino	7.471	47,9	3.228 (43,2)	2.728 (36,5)	
Faixa etária					< 0,001
< 1 ano	2.841	18,2	1.112 (39,1)	1.050 (36,9)	
1 a 4 anos	2.283	14,6	964 (42,2)	879 (38,5)	
5 a 11 anos	499	3,2	222 (44,4)	192 (38,4)	
12 a 17 anos	677	4,3	300 (44,3)	226 (33,3)	
30 a 49 anos	2.678	17,2	994 (37,1)	1.048 (39,1)	
18 a 29 anos	6.190	39,7	2.556 (41,2)	2.579 (41,6)	
50 anos ou mais	430	2,8	154 (35,8)	205 (47,6)	
Escolaridade^c^					< 0,001
< 4 anos	198	5,6	87 (43,9)	85 (42,9)	
4 a 9 anos	852	24,1	281 (32,9)	438 (51,4)	
10 a 12 anos	1.987	56,1	695 (34,9)	947 (47,6)	
13 anos ou mais	504	14,2	224 (44,4)	188 (37,3)	
Raça/cor da pele					< 0,001
Branco	9.246	70,7	4.046 (43,7)	3.566 (38,5)	
Preto	427	3,3	156 (36,5)	198 (46,7)	
Pardo	3.241	24,8	1.195 (36,8)	1.505 (46,4)	
Amarelo	130	1,0	53 (40,7)	53 (40,7)	
Indígena	37	0,3	14 (37,8)	18 (48,6)	
Origem da transmissão^d^					0,02
Importado	33	0,3	20 (60,6)	10 (30,3)	
Autóctone – ESP	12.832	99,7	5.308 (41,3)	5.076 (39,5)	
Evolução					0,04
Cura	13.019	99,9	5.387 (41,3)	5.225 (40,1)	
Óbito	17	0,1	3 (17,6)	10 (58,8)	

Fonte: Sinan, 2019.

^a^ Exluídos os casos com informação ignorada.

^b^ Frequências absolutas e relativas.

^c^ Apenas casos com 18 anos ou mais de idade.

^d^ Foram considerados autóctones do estado de São Paulo, SP.

Brancos compuseram a maior parcela dos casos de sarampo, o equivalente a 70,7% (9.246), seguidos de pardos 24,8% (3.241), pretos 3,3% (427), amarelos (asiáticos) 1% (130), e indígenas 0,3% (37) (Tabela 2).

A maioria dos casos (12.832; 99,9%) foi originada por transmissão autóctone no próprio estado de São Paulo. Dentre os importados, a maior parte (20/33) era procedente de outros estados do país, sendo os demais (13/33) viajantes internacionais (Tabela 2).

Foi observada diferença significativa (p < 0,001) nas proporções de vacinação prévia entre casos femininos (43,2%) e masculinos (37,9%). Em relação à idade, as frequências de vacinação prévia foram maiores nas faixas etárias mais jovens, quando comparadas às faixas mais idosas (p < 0,001). Quanto à escolaridade, as maiores proporções de vacinação prévia foram observadas nas duas categorias extremas, ou seja, no grupo com menos de quatro anos e no grupo com mais de 13 anos de estudo (p < 0,001) (Tabela 2).

Em relação à raça/cor da pele registrada, foi observada maior proporção de vacinação entre brancos (43,7%) quando comparada a pretos (36,5%), pardos (36,8%), amarelos (40,7%) e indígenas (37,8%) (p < 0,001). De forma semelhante, observou-se maior proporção de vacinação prévia entre os casos importados (60,6%), quando comparado aos casos autóctones (41,3%) (p = 0,02), assim como entre os que evoluíram para cura (41,3%), quando comparado aos que evoluíram para óbito (17,6%) (p = 0,04) (Tabela 2).

Além de febre e rash cutâneo, tosse e coriza foram os sinais mais frequentes e estiveram presentes em 79,1% (11.871) e 65,4% (9.779) dos casos, respectivamente. Em contrapartida, a conjuntivite foi menos frequente e esteve presente em 34,9% (5.144) dos casos confirmados de sarampo em 2019. Quando estratificados por situação vacinal, tosse e conjuntivite foram significativamente mais frequentes entre casos não vacinados quando comparado aos casos que referiram vacinação prévia (p < 0,001).

Em relação à incidência de hospitalização, observou-se maior risco (RI = 2,19; IC95% 1,66–2,88) em crianças menores de um ano, quando comparado à faixa etária mais idosa (50 anos e mais) (Tabela 3). Adicionalmente, observou-se maior risco de hospitalização nos casos não vacinados quando comparado aos que relataram vacinação anterior (RI = 1,59; IC95% 1,45–1,75) (Tabela 3).

**Tabela 3:** Incidência de hospitalização segundo características epidemiológicas e socioeconômicas dos casos de sarampo. Estado de São Paulo, Brasil, 2019.

Variáveis	Total de casos^a^	Hospitalização		RI	IC95%
		n	Incidência^a^		
Sexo					
Masculino	8.123	1.086	13,4	1	
Feminino	7.471	952	12,7	0,95	(0,87–1,03)
Faixa etária					
50 ou mais	430	47	10,9	1	
30 a 49	2.678	279	10,4	1,03	(0,77–1,38)
18 a 29	6.190	601	9,7	0,90	(0,68–1,19)
12 a 17	677	68	10,0	0,92	(0,65–1,30)
5 a 11	499	50	10,0	0,93	(0,64–1,36)
1 a 4	2.283	304	13,3	1,22	(0,92–1,63)
< 1	2.841	690	24,3	2,19	(1,66–2,88)
Escolaridade ^b^					
< 4 anos	198	10	5,1	1	
4 a 9 anos	852	44	5,2	1,07	(0,55–2,08)
10 a 12 anos	1.987	118	5,9	1,20	(0,64–2,24)
13 anos ou mais	504	45	8,9	1,14	(0,58–2,25)
Raça/cor registrada					
Branco	9.246	1.168	12,6	1	
Preto	427	44	10,3	0,82	(0,62–1,09)
Pardo	3.241	389	12,0	0,96	(0,87–1,07)
Amarelo	130	17	13,1	1,01	(0,64–1,56)
Indígena	37	8	21,6	1,84	(1,01–3,34)
Vacinação prévia					
Sim	6.302	642	10,2	1	
Não	6.179	1.004	16,2	1,59	(1,45–1,75)

Fonte: Sinan, 2019.

RI: razão de incidência; IC95%: intervalo de confiança de 95%.

^a^ Excluídos os casos com informação ignorada.

^b^ Apenas casos com 18 anos ou mais de idade.

## DISCUSSÃO

Os achados do presente estudo propiciam reflexões importantes sobre a maior epidemia de sarampo do Brasil no período pós-eliminação. Em relação à origem da epidemia, os dados secundários não permitiram uma conclusão definitiva. Embora não haja um detalhamento sobre a origem do primeiro caso notificado no estado de São Paulo em 2019, supõe-se que ele não seja o caso índice, por ser tratar de um lactente de seis meses de idade, classificado como autóctone. Em um cenário de eliminação da transmissão, é possível que os primeiros casos sejam confundidos com outras infecções virais exantemáticas inespecíficas. Além disso, as manifestações clínicas em indivíduos vacinados (ainda que com esquemas incompletos) tendem a se apresentar de forma mais branda, o que pode levar à subnotificação^9,17,18^.

Os achados do presente estudo corroboram esse fato ao indicarem maior risco de hospitalização entre casos não vacinados, quando comparado aos vacinados, assim como as diferenças observadas na própria apresentação clínica da doença, com menor frequência de tosse e conjuntivite entre vacinados, quando comparado aos casos sem histórico de vacinação.

O número considerável de casos da doença entre indivíduos com histórico de vacinação prévia é um dado preocupante que pode sugerir perda da efetividade da vacina ou ainda limitações no registro dessa informação nos sistemas de notificação. Alguns estudos têm relatado o desenvolvimento da doença em pessoas que foram vacinadas com as duas doses na infância. Algumas razões possíveis incluem a redução dos níveis de anticorpos com o passar do tempo e perda de efetividade devido a problemas na cadeia de frios^19^. Há ainda evidências de que diferenças entre o genótipo do vírus utilizado na vacina e o genótipo das cepas circulantes também possam contribuir para o desenvolvimento da doença em indivíduos vacinados^23,24^.

Em relação à qualidade do registro sobre a vacinação, embora haja orientação técnica sobre a checagem da caderneta de vacinação dos casos, é mais provável que essa informação na rotina dos serviços assistenciais seja autorreferida. Adicionalmente, é importante salientar que a ficha de notificação não especifica se o caso tinha esquema vacinal completo com duas doses. Dessa forma, é possível que parte dos casos da doença entre indivíduos com vacinação prévia na epidemia de São Paulo seja consequência de esquemas vacinais incompletos, com consequente redução da eficácia da vacina. O maior acometimento de adultos jovens na epidemia de São Paulo reforça essa hipótese, uma vez que essa faixa etária tem sido apontada em outros estudos como a mais propensa a ter esquema vacinal incompleto^4,25^.

As crianças menores de cinco anos foram a segunda faixa etária mais atingida pela doença e, junto com adultos jovens, compuseram o perfil etário predominante da epidemia de São Paulo. Estes achados corroboram a tendência observada de aumento da morbidade por sarampo em adultos jovens, antes restrita às crianças^4^.

Se por um lado há indícios de falhas na imunização ou mesmo de esquemas vacinais incompletos, por outro, a análise dos dados também apontou um expressivo número de casos sem vacinação prévia, o que, em todo caso, indica baixa performance dos programas de imunização de rotina ou deficiência no registro dessa informação. A formação de *clusters* de populações suscetíveis decorrentes de coberturas vacinais inadequadas pode funcionar como iniciadores de surto em regiões em fase de eliminação da doença, conforme já demonstrado em estudos anteriores^9,18^.

Em crianças, há tendência de redução da cobertura vacinal com o aumento da idade, principalmente em relação às vacinas administradas em doses múltiplas, como é o caso da tríplice viral contendo sarampo. No primeiro ano de vida, a criança recebe uma grande quantidade de vacinas do calendário de rotina, seguido de um intervalo de meses ou anos, o que gera uma sensação de tranquilidade que pode contribuir para o esquecimento de pais e cuidadores^31,32^. Importante lembrar que no Brasil, a segunda dose da tríplice viral, que antes era recomendada aos 4–6 anos, passou a ser antecipada para os 15 meses em 2013^33^. Esse fato pode explicar, ao menos em parte, a maior frequência de casos em adultos jovens e em coortes de nascimentos mais antigas. Cabe lembrar que a vacina contra o sarampo é preconizada até os 49 anos de idade, pois presume-se que acima dessa idade a pessoa já teve contato natural com o vírus, tendo assim imunidade duradoura.

Os achados também revelaram menor proporção de vacinação em grupos menos escolarizados quando comparados aos de maior escolaridade (13 ou mais anos de estudo), assim como em indivíduos pretos e pardos quando comparados a brancos, achado que sugere acesso desigual à vacinação^34^. O fato de 70% dos casos terem sido identificados como brancos pode estar relacionado ao maior poder aquisitivo e possibilidades de viagens para locais com circulação do vírus e, como consequência, maior probabilidade de infecção de pessoas próximas no retorno.

Quanto à análise da difusão espacial, os achados revelaram dois padrões principais. O primeiro, caracterizado por um processo de difusão por contiguidade, no qual a partir do polo pioneiro (regiões metropolitanas de São Paulo e Baixada Santista) se estabeleceu três eixos principais que conectam municípios próximos localizados nas regiões intermediária de Campinas, São José dos Campos e Sorocaba. O segundo padrão se caracteriza pela relação do polo pioneiro com municípios mais distantes, pertencentes às regiões intermediárias de Ribeirão Preto e São José do Rio Preto, por meio de um processo de difusão hierárquica por realocação.

Ambos os padrões de difusão do sarampo refletem as características estruturais do território, seguindo trajetórias semelhantes às das principais rodovias do estado e da hierarquia urbana de regiões de influência das cidades em relação à centralidade na distribuição de bens, equipamentos, serviços e mobilidade populacional. Sob esse aspecto, ressalta-se o papel da grande São Paulo (Metrópole Nacional), das capitais regionais Campinas, São José dos Campos, Sorocaba, Ribeirão Preto, Santos, Limeira, e dos centros sub-regionais Itapetinga, Fernandópolis e Registro. Além de polo pioneiro e principal difusor de casos para as outras regiões, cabe destacar que a região da Grande São Paulo manteve altas incidências durante a maior parte da epidemia em 2019.

Esses achados reforçam a importância da hierarquia urbana no processo de difusão de doenças com transmissão semelhante à do sarampo, a exemplo de estudos recentes realizados para covid-19 nos estados de São Paulo^35^ e do Rio de Janeiro^36^. A incorporação de outras informações do território não utilizadas neste estudo, a exemplo de fluxos de pessoas em rotas aéreas e rodoviárias, podem contribuir para melhor caracterização dos processos envolvidos na difusão de sarampo e subsidiar ações de vigilância no âmbito intermunicipal.

As limitações deste estudo estão relacionadas principalmente à qualidade da informação oriunda do sistema de informação. No caso do registro de vacinação prévia, é provável que a informação seja autorreferida na maioria dos casos, apesar da orientação de checagem da caderneta de vacinação. Já a variável raça/cor da pele, embora seja um campo da ficha de notificação destinado a autoidentificação, é relativamente comum na rotina dos serviços assistenciais que ele seja preenchido pelo próprio profissional que fez o atendimento do caso. Nesse sentido, a depender das percepções dos que coletam essa informação, a mensuração das iniquidades de acesso a partir dessa variável podem ser distorcidas.

Em última análise, os resultados reforçam a necessidade de priorização nas estratégias de imunização dos subgrupos mais acometidos, formado por adultos jovens, para além da vacinação de rotina em crianças. Adicionalmente, estudos que busquem investigar a ocorrência de *clusters* de populações vulneráveis, propensas a menor cobertura de vacinação, são essenciais para ampliar a compreensão sobre a dinâmica de transmissão da doença e assim reorientar estratégias de controle mais eficazes para garantir a manutenção da eliminação da doença.
